# Decreased Responsiveness to Chemical Itch in Old Mice

**DOI:** 10.3390/cells14120889

**Published:** 2025-06-12

**Authors:** Qiaofeng Zhao, Mitsutoshi Tominaga, Sumika Toyama, Kotaro Honda, Eriko Komiya, Yayoi Kamata, Hang Ma, Kenji Takamori

**Affiliations:** 1 Juntendo Itch Research Center (JIRC), Institute for Environmental and Gender-Specific Medicine, Juntendo University Graduate School of Medicine, Chiba 279-0021, Japan; zhao@juntendo.ac.jp (Q.Z.); tominaga@juntendo.ac.jp (M.T.); su-toyama@juntendo.ac.jp (S.T.); kt-honda@juntendo.ac.jp (K.H.); ekomiya@juntendo.ac.jp (E.K.); ykamata@juntendo.ac.jp (Y.K.); 2 Department of Functional Morphology, Faculty of Pharmacy, Juntendo University, Chiba 279-0013, Japan; 3 Department of Biomedical and Pharmaceutical Sciences, College of Pharmacy, University of Rhode Island, Kingston, RI 02881, USA; hang_ma@uri.edu; 4 Department of Dermatology, Juntendo University Urayasu Hospital, Chiba 279-0021, Japan

**Keywords:** aging, calcium imaging, Cav3.2, dorsal root ganglion, pruritus, TRPV1, itch mechanisms

## Abstract

Aging is associated with altered itch perception, potentially due to changes in neuronal function and pruriceptive signaling. The underlying mechanisms, however, remain unclear. We investigated age-related differences in itch sensitivity at behavioral, cellular, and molecular levels. Young and old mice were intradermally injected with various pruritogens, including small molecules (histamine, chloroquine, and serotonin) and peptides (BAM8–22, AY-NH_2_, and SLIGRL-NH_2_). Scratching behavior and mechanical itch sensitivity were assessed, and calcium imaging was used to evaluate sensory neuron responses in the dorsal root ganglia. Additionally, immunofluorescence staining was performed to analyze the expression of TRPV1 and Cav3.2. Old mice exhibited reduced scratching behavior following injections, and their neuronal responses to histamine and chloroquine were diminished. Although all treated groups showed increased mechanical alloknesis, the effect was less pronounced in old animals. The expression of TRPV1 and Cav3.2 was also reduced in dorsal root ganglia neurons of old mice. These findings suggest that aging impairs both functional responsiveness and molecular signaling in sensory neurons, contributing to reduced chemical itch sensitivity in aged individuals.

## 1. Introduction

Itch, or pruritus, is a relatively common complaint across all age groups and can significantly impair quality of life, particularly in older adults [1]. While itch is often associated with allergic or immune responses, it can also result from neuropathic and psychogenic causes [2]. Recent research has identified molecular receptors, such as transient receptor potential (TRP) channels, that contribute to neuronal activation in itch pathways [3]. In the elderly, epidermal changes such as elevated surface pH may further influence itch perception [4]. Although the signaling pathways involved in pruritus have been extensively studied in animal models, the effects of aging on itch behavior and neuronal signaling remain unclear.

Pruritogenic small molecules (e.g., histamine, chloroquine, and serotonin) and peptides, including a sensory neuron-specific receptor agonist (bovine adrenal medulla 8–22; BAM 8–22) and selective protease-activated receptor-4 and -2 agonists (AY-NH_2_ and SLIGRL-NH_2_, respectively), activate distinct receptors and directly stimulate sensory C-fibers, thereby contributing to chemical itch sensations. [5]. However, the association of age with the pathophysiological mechanisms underlying pruritogen-induced pruritus remains unclear and insufficiently explored, and investigating pruritogen-induced pruritus is a useful approach to better understanding the mechanisms of itch.

Itch signals are transmitted from peripheral sensory nerve endings to dorsal root ganglia (DRG) neurons via receptors, including transient receptor potential vanilloid 1 (TRPV1) and Mas-related G protein-coupled receptors (Mrgprs), which then relay signals to the spinal cord and brain [6]. TRPV1, a non-selective cation channel, is well known for mediating noxious heat, inflammatory pain, and pruritus [7,8,9]. It is also sensitized by inflammatory mediators, thus enhancing neuronal excitability, and its involvement in histaminergic itch has been reported [10]. Cav3.2, a subtype of calcium channels, is primarily expressed in DRG neurons and involved in action potential generation and propagation during itch and pain signaling. It mediates both histaminergic and non-histaminergic itch responses [11]. Yet whether its expression or function changes with age remains unclear.

In this study, we assessed the effects of aging on pruritogen-induced itch behaviors, DRG neuronal activity, and the expression of TRPV1 and Cav3.2. By comparing young and old mice, we aimed to identify age-related changes in peripheral itch mechanisms and potential molecular targets for managing pruritus in the elderly.

## 2. Materials and Methods

### 2.1. Animals

Male C57BL/6 J mice aged 8–16 (young) and 65–80 (old) weeks were obtained from Oriental Yeast (Tokyo, Japan). They were housed under a 12 h light/dark cycle at a controlled temperature of 22–24 °C, with access to food and water ad libitum, in the experimental animal facility at Juntendo University Graduate School of Medicine. All animal procedures were approved by the Animal Care and Use Committee of Juntendo University Graduate School of Medicine and conducted following the National Institutes of Health guidelines for the care and use of laboratory animals, with every effort made to minimize animal discomfort and distress.

### 2.2. Behavioral Treatment and Measurements

#### 2.2.1. Intradermal Injection of Pruritogens and the Itch-Induced Scratch Counting Assay

Young (*n* = 84) and old (*n* = 84) mice were randomly assigned to six itch pruritogen treatment groups. Two days after shaving their backs, mice received intradermal injections of one of the following compounds: histamine (50 μg/10 μL, H7250 from Sigma-Aldrich, Saint Louis, MO, USA), chloroquine (CQ, 100 μg/10 uL, C6628 from Sigma-Aldrich, Saint Louis, MO, USA), serotonin (8.28 μg/10 μL, 3547 from Tocris, Bristol, UK.), BAM (8–22) (100 μg/10 μL, 1763 from Tocris, Bristol, UK), AY-NH_2_ (100 μg/10 μL, 1487 from Tocris, Bristol, UK), and SLIGRL-NH_2_ (100 μg/10 μL, 1468 from Tocris, Bristol, UK.) as described [12]. The control group received saline. Immediately after injections, scratching behavior was recorded for 2 h using the SCLABA^®^-Next system (Noveltec, Kobe, Japan) to assess itch-related responses [13].

#### 2.2.2. Mechanical Alloknesis Assay

A mechanical alloknesis assay was conducted following a standard protocol [14]. Briefly, starting 30 min after intradermal injection of the pruritogen into the shaved backs, von Frey filaments with bending forces of 0.07 g and 0.16 g were applied to the backs of young (*n* = 48) and old (*n* = 48) mice. Each von Frey filament was applied for 1 s with 2 to 5 s intervals between applications, totaling 30 stimulations administered around the injection site. Naïve mice (young, *n* = 8; old, *n* = 8) served as controls and received identical filament applications without prior injection.

#### 2.2.3. Mechanical Itch Assay

A mechanical itch assay was performed in young (*n* = 24) and old (*n* = 24) mice following a standard protocol [15]. As in the mechanical alloknesis assay, von Frey filaments with bending forces of 0.07 g and 0.16 g were applied to the post-auricular region (behind the ear) starting 30 min after intradermal injection of the pruritogen into the shaved back skin to assess mechanically induced itch responses.

### 2.3. DRG Neuron Cultures

DRG were isolated from the spinal columns of C57BL/6 mice under sterile conditions and enzymatically dissociated using collagenase (LS004176 from Worthington, Columbus, Ohio, USA), L-cysteine (C7352-25 from Sigma-Aldrich, Saint Louis, MO, USA), and papain (LS003126 from Worthington, Columbus, Ohio, USA). The dissociated neurons were then plated onto poly-D-lysine (PDL) (A3890401 from Thermo Fisher, Waltham, MA, USA) and laminin (23017015 from Thermo Fisher, Waltham, MA USA)-coated culture dishes or glass coverslips to promote cell adhesion. Cells were maintained in complete culture medium (CC-4461 from Lonza, Switzerland) at 37 °C in a humidified incubator with 5% CO_2_ until used in experiments [16].

### 2.4. Calcium Imaging

DRG neurons from young (*n* = 6) and old (*n* = 5) mice cultured on coverslips were loaded with the ratiometric calcium indicator Fura-2 using theFura-2 Calcium Kit (341-91271 from Dojindo, Kumamoto, Japan) for 1 h at 37 °C in a humidified incubator with 5% CO_2_, following the manufacturer’s instructions. During imaging, cells were perfused with Ringer’s solution containing 140 mM NaCl, 4 mM KCl, 2 mM CaCl_2_, 1 mM MgCl_2_, 10 mM HEPES, and 4.54 mM NaOH, adjusted to pH 7.4. A glucose-supplemented version contained an additional 5 mM glucose for the first wash.

Calcium imaging was performed using confocal laser-scanning microscopy (TCS SP5, Leica Microsystems, Wetzlar, Germany). Intracellular Ca2^+^ dynamics were recorded by measuring Fura-2 fluorescence ratios (F340/F380) in response to histamine (100 µM) and chloroquine (CQ, 100 µM). A calcium response was defined as a peak fluorescence increase exceeding 20% of baseline. Neuronal viability was confirmed in each experiment by depolarization with high-potassium Ringer’s solution (50 mM KCl), which consistently evoked Ca2^+^ transients [17].

### 2.5. Immunofluorescence Staining of Cultured DRG Neurons

Cultured DRG neurons from young (*n* = 3) and old (*n* = 3) mice were incubated for 24–36 h at 37 °C in a humidified atmosphere containing 5% CO₂. Cells were then fixed with 4% paraformaldehyde, permeabilized with 0.1% Triton X-100, and blocked with a solution containing 2% bovine serum albumin and 5% normal donkey serum. Neurons were incubated with primary antibodies against TRPV1 (1:200; ACC-030 from Alomone Labs, Jerusalem, Israel) and Cav3.2 (1:200; ACC-025 from Alomone Labs, Jerusalem, Israel) for 2 h at room temperature or overnight at 4 °C. After washing, cells were incubated with species-appropriate Alexa Fluor-conjugated secondary antibodies: donkey anti-rabbit Alexa Fluor 594 (1:300; A-21207 from Invitrogen, Waltham, MA, USA) for TRPV1 and goat anti-rabbit Alexa Fluor 488 (1:300; A-11008 from Invitrogen, Waltham, MA, USA) for Cav3.2. Nuclei were counterstained with DAPI. Images were captured using a fluorescence microscope (Keyence bz-x800; Osaka, Japan). TRPV1/Cav3.2 c-Fos-positive cells were quantified using Fiji (Fiji Is Just ImageJ, based on ImageJ 1.54g) software, and the protein expression area relative to nuclear area was measured. Data were collected from three independent cultures, with multiple randomly selected regions (≥3 per dish) analyzed per culture dish.

### 2.6. Statistical Analysis

All experiments were repeated at least three times. Data are presented as means ± standard error of the means. Statistical analyses were performed using GraphPad Prism 9 (GraphPad Software, San Diego, CA, USA). Comparisons were made using *t*-tests, permutation tests, or two-way ANOVA. A *p*-value of less than 0.05 was considered statistically significant and expressed as the mean ± SEM.

## 3. Results

### 3.1. Aging Alters Scratching Behavior Following Itch Mediator Administration

**Figure 1:**
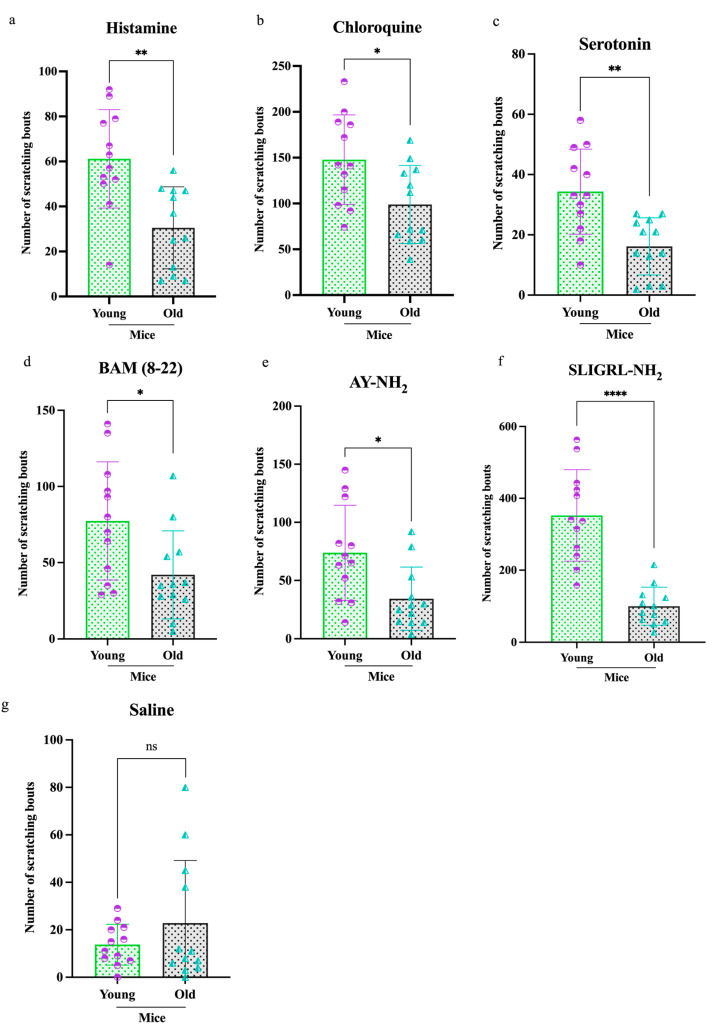
Differential scratching responses to pruritogens in old and young mice. Old mice (total *n* = 84) exhibited significantly less scratching over a 2 h period following intradermal injection of the following pruritogens compared to young mice (total *n* = 84): (**a**) histamine (**p < 0.005), (**b**) chloroquine (*p < 0.05), (**c**) serotonin (**p < 0.005), (**d**) bovine adrenal medulla 8–22 (BAM 8–22) (*p < 0.05), (**e**) AY-NH_2_ (*p < 0.05), (**f**) SLIGRL-NH_2_ (****p < 0.0001), and (**g**) saline (ns). Mice were randomly assigned to six pruritogen treatment groups. For each assay, eight mice were used (young = 4, old = 4), and each pruritogen experiment was independently repeated three times. Comparisons were made using *t*-tests.

To examine age-related differences in itch behavior, young and old mice were injected intradermally with one of six pruritogens, and scratching responses were recorded over a 2 h period using the SCLABA^®^-Next system. Across all treatments, old mice (*n* = 84) exhibited significantly reduced scratching behavior compared to young mice (*n* = 84) (Figure 1). Specifically, older mice showed decreased responses to (a) histamine (**p < 0.01), (b) CQ (*p < 0.05), (c) serotonin (**p < 0.01), (d) BAM (8–22) (*p < 0.05), (e) AY-NH_2_ (*p < 0.05), (f) SLIGRL-NH_2_ (****p < 0.0001), and (g) saline (ns). Among these, SLIGRL-NH_2_ elicited the most significant age-related difference in scratching behavior, followed by histamine, serotonin, CQ, BAM (8–22), and AY-NH_2_.

### 3.2. Pruritogen-Induced Mechanical Alloknesis Increases Without Affecting Mechanical Itch

**Figure 2:**
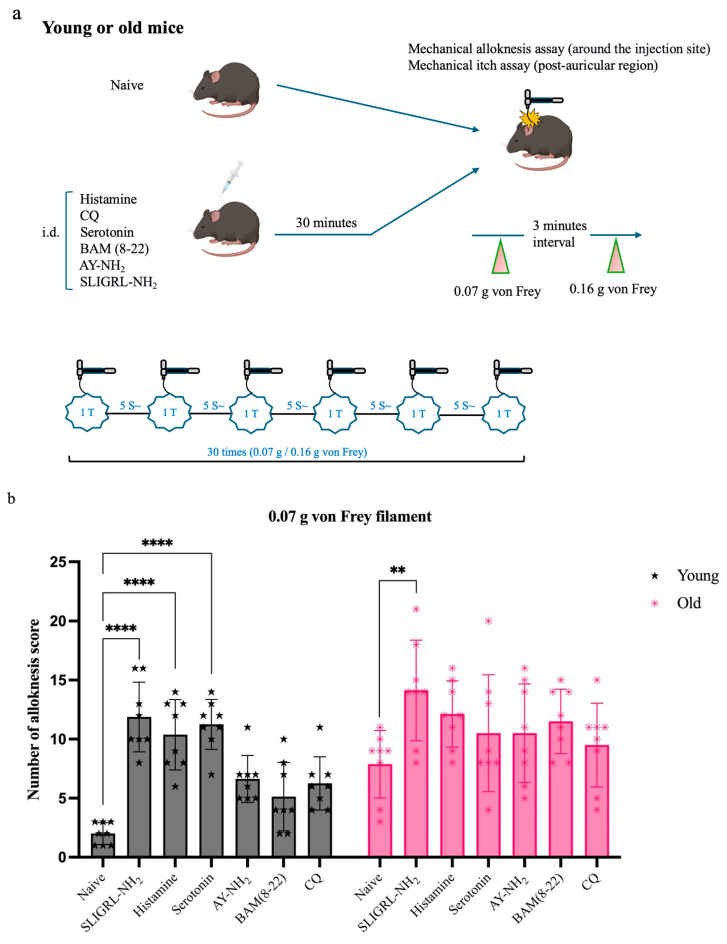

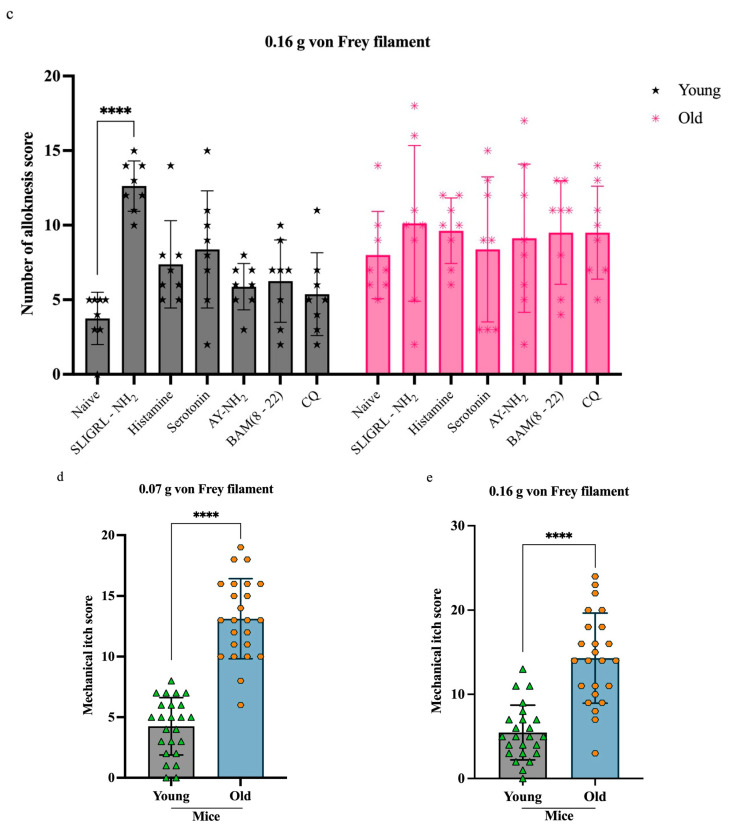
Response to pruritogens in mechanical alloknesis and mechanical itch. (**a**) Each group of mice underwent mechanical alloknesis (back) (young = 48 and old *n* = 48) or mechanical itch (behind the ear) (young *n* = 24 and old *n* = 24) assays using 0.07 g or 0.16 g von Frey filaments 30 min after intradermal injection of pruritogens (**b**,**c**). Compared to naïve mice, alloknesis scores increased in both old and young groups. In the old group, SLIGRL-NH_2_ significantly increased alloknesis scores at 0.07 g (** p < 0.005). In the young group, SLIGRL-NH_2_, histamine, and serotonin significantly increased alloknesis scores at 0.07 g (**** p < 0.0001), with SLIGRL-NH_2_ also showing a significant effect at 0.16 g (**** p < 0.0001). (**d**,**e**) For the mechanical itch assay, naïve and pruritogen-injected groups were analyzed separately, and no significant differences were found within each age group. With data pooled for analysis, a significant difference in mechanical itch responses between old and young mice was observed for both 0.07 g and 0.16 g von Frey filament stimulations (**** p < 0.0001) and 0.16 g (**** p < 0.0001). Mice were randomly assigned to six pruritogen treatment groups. For each assay, four mice were used (young *n* = 2, old *n* = 2), and each pruritogen experiment was independently repeated at least three times. Comparisons were made using two-way ANOVA (a,b) and *t*-tests (**c**,**d**).

To assess change in mechanical hypersensitivity, we evaluated mechanical alloknesis 30 min after intradermal pruritogen injection (Figure 2a). All pruritogen-treated groups showed significantly elevated alloknesis scores compared to their respective naïve controls (young *n* = 8, old *n* = 8). However, this increase was less pronounced in old mice (*n* = 48). At the 0.07 g filament force level (Figure 2b), SLIGRL-NH_2_ induced significant alloknesis in both old (**p < 0.005) and young mice (****p < 0.0001), whereas histamine and serotonin elicited significant responses only in young mice (****p < 0.0001). At the 0.16 g stimulus level (Figure 2c), the effect of SLIGRL-NH_2_ remained effective in young mice (****p < 0.0001) but was no longer significant in old mice (Figure 2b, c).

In contrast, mechanical itch scores showed no significant differences between naïve (young *n* = 8, old *n* = 8) and pruritogen-treated (young *n* = 16, old *n* = 16) mice within each age group. Therefore, data from both conditions were pooled to evaluate age effects. Overall, mechanical itch sensitivity was significantly higher in old mice compared to young mice (Figure 2d,e, ****p < 0.0001).

### 3.3. Reduced Pruritogen-Induced Ca^2+^ Responses in DRG Neurons of Old Mice

**Figure 3:**
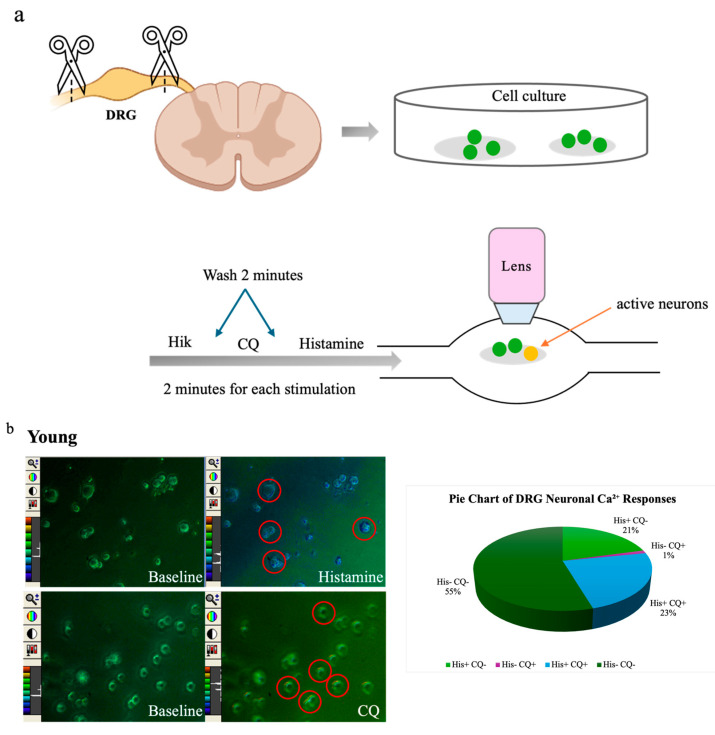

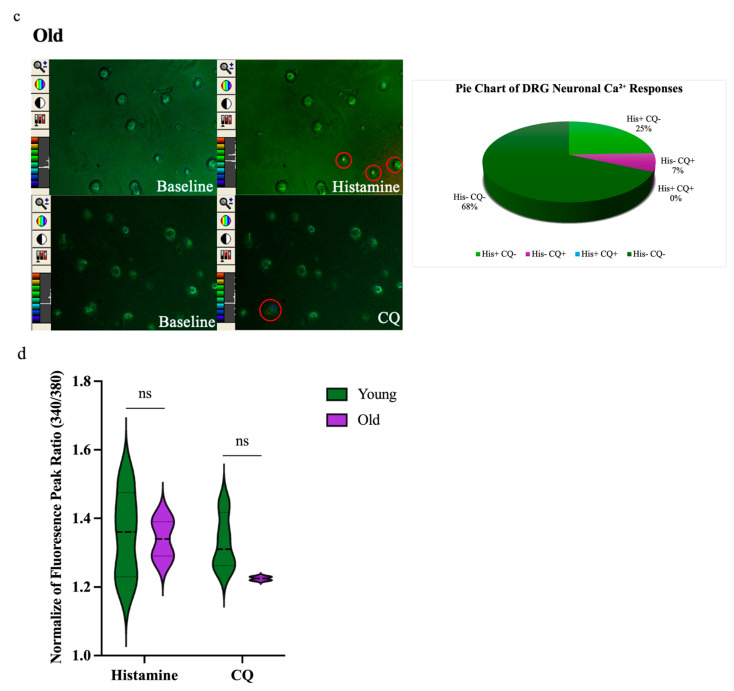
Ca^2+^ response to pruritogens was reduced in dorsal root ganglion (DRG) neurons of old mice. (**a**) Confocal laser-scanning microscopy images show DRG neurons from young (*n* = 6) and old (*n* = 5) mice. (**b**,**c**) Multiple DRG neurons exhibited reactivity to histamine and CQ exposure. Representative single-frame images show spectral color changes in DRG neurons corresponding to variations in the 340/380 fluorescence ratio (20× magnification). An increase in the 340/380 ratio indicates a transient rise in intracellular calcium, reflecting neuronal activation. Pie charts depict the distribution of DRG neuron responsiveness to histamine and CQ stimulation. Neurons were categorized based on calcium responses into four groups: responsive to histamine only (His+ CQ–): 21% in young and 25% in old mice; to CQ only (His– CQ+): 1% in young and 7% in old mice; to both (His+ CQ+): 23% in young and 0% in old; or to neither (His– CQ–): 55% in young and 68% in old. (**d**) Violin plots suggested a difference in fluorescence peak ratios; however, statistical analysis showed no significant differences (ns) between young and old mice in response to either histamine or CQ. Each assay was conducted using two mice (young *n* = 1, old *n* = 1). The dissociated neurons, which varied in number, were randomly plated onto glass coverslips. Comparisons were made using a permutation test.

To further explore age-related differences in sensory neuron activity, we performed calcium imaging assays on cultured DRG neurons from young (*n* = 6, 322 neurons) and old (*n* = 5, 200 neurons) mice. Neurons were sequentially stimulated with histamine (100 µM), chloroquine (100 µM), and high potassium (HiK, 50 mM) (Figure 3a). Pruritogen-induced calcium influx was visualized as changes in Fura-2 fluorescence ratios, which had color shifts from green to blue, yellow, or red. A smaller proportion of DRG neurons from old mice responded to HiK (41%, 82/200) compared to those from young mice (54%, 174/322), indicating a general reduction in excitability. Among HiK-responsive neurons, the percentage responding to histamine was 44% (77/174) in young mice vs. 24% (20/82) in old mice, and the percentage responding to chloroquine was 25% (43/174) in young mice vs. 7% (6/82) in old mice. Neurons were categorized based on response profiles: histamine-only (His^+^CQ^−^), CQ-only (His^−^CQ^+^), both (His^+^CQ^+^;), or neither (His^−^CQ^−^). In young mice, 21% responded to histamine only, 1% to chloroquine only, 23% to both, and 55% to neither. In old mice, 25% responded to histamine only, 7% to chloroquine only, 0% to both, and 68% to neither (Figure 3b,c). Although peak calcium response amplitudes to histamine and chloroquine trended lower in old neurons, these differences were not statistically significant (Figure 3d).

### 3.4. Reduced TRPV1 and Cav3.2 Expression in DRG Neurons of Old Mice

**Figure 4:**
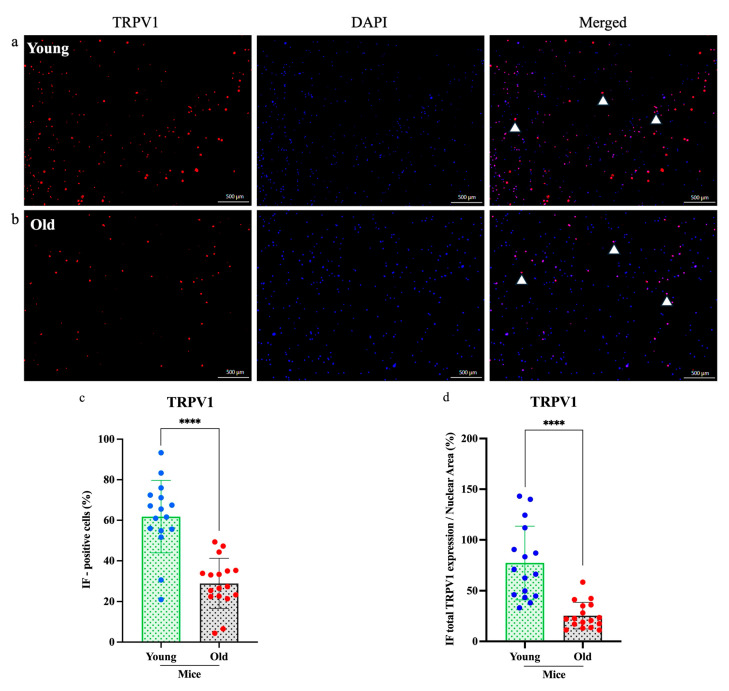
Immunofluorescence analysis of TRPV1 expression in dorsal root ganglia neurons. (**a**,**b**) Expression of transient receptor potential vanilloid 1 (TRPV1) (red) in old mice was significantly lower than in young mice, as shown in the immunofluorescence images. (**c**,**d**) Both the percentage of immunofluorescence-positive cells and the total TRPV1 expression-to-nuclear area ratio were markedly reduced in old mice. Cell counts were obtained from three independent cultures, with more than three regions analyzed per dish (*n* = 3, ****p < 0.0001, 4× magnification). Each assay was conducted using two mice (young *n* = 1, old *n* = 1) and was independently repeated a minimum of three times. Comparisons were made using a *t*-test.

**Figure 5:**
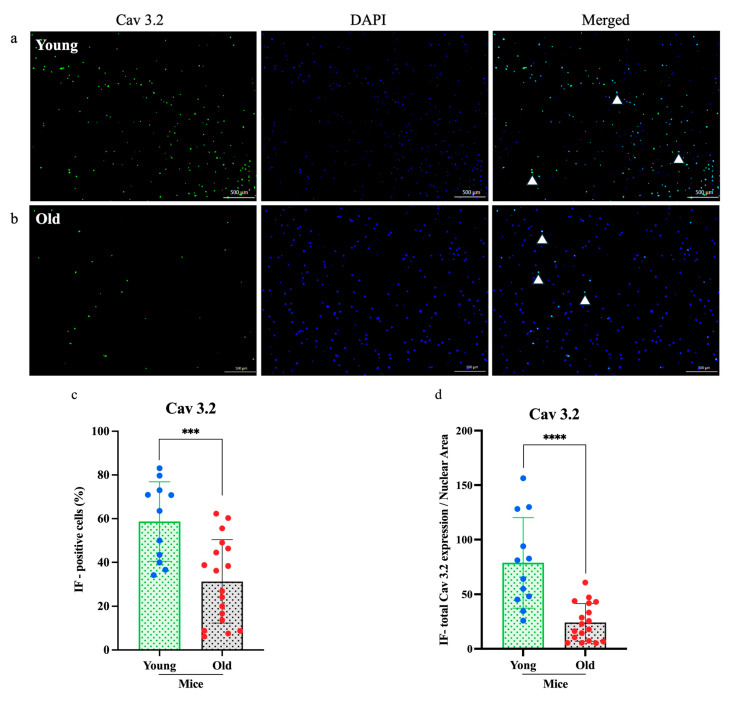
Immunofluorescence analysis of subtype calcium channel Cav 3.2 expression in dorsal root ganglia neurons. (**a**,**b**) The expression of Cav 3.2 (green) in old mice was significantly lower than in young mice, as shown in the immunofluorescence images. (**c**,**d**) Both the percentage of immunofluorescence-positive cells and the total Cav 3.2 expression-to-nuclear area ratio were markedly reduced in old mice. Cell counts were obtained from three independent cultures, with more than three regions analyzed per dish (*n* = 3, ***p < 0.0005, ****p < 0.0001, 4× magnification). Each assay was conducted using two mice (young *n* = 1, old *n* = 1) and was independently repeated a minimum of three times. Comparisons were made using *t*-tests.

To determine whether aging affects the molecular profile of sensory neurons, we examined TRPV1 and Cav3.2 expression in cultured DRG neurons from young (*n* = 3) and old (*n* = 3) mice using immunofluorescence staining (Figure 4 and Figure 5). Fluorescence microscopy revealed markedly lower expression of both proteins in neurons from old mice compared to young mice. Quantitative analysis revealed a significant reduction in the numbers of TRPV1-positive neurons (****p < 0.0001; Figure 4c) and Cav3.2-positive neurons (*** *p* < 0.0005; Figure 5c). Furthermore, the relative expression areas (TRPV1/nuclear and Cav3.2/nuclear) were significantly decreased in old neurons (****p < 0.0001 for both; Figure 4d and Figure 5d), indicating an age-related decline in sensory neuron protein expression.

## 4. Discussion

**Figure 6:**
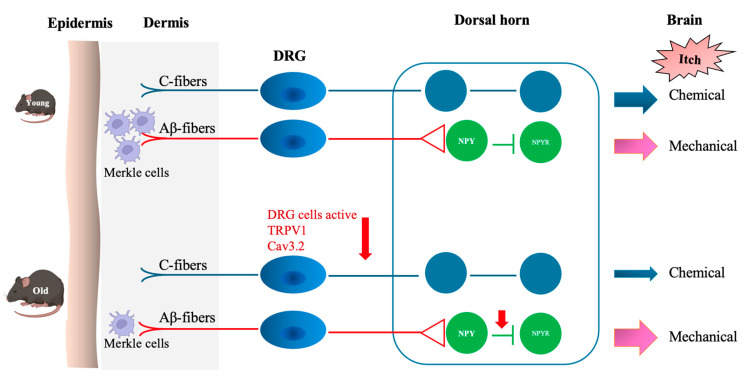
Chemical and mechanical itch transduction pathways. Chemical itch is mediated by pruritogens that activate distinct receptors and directly stimulate peripheral sensory C-fibers, triggering chemical itch signaling. In old mice, diminished responsiveness of dorsal root ganglion (DRG) neurons—along with reduced expression of TRPV1 and Cav3.2 channels—leads to decreased chemical itch sensitivity. Mechanical itch by light mechanical stimuli activates Aβ low-threshold mechanoreceptors (Aβ-LTMRs), which then excite spinal interneurons expressing neuropeptide Y receptor 1 (NPY1R). Normally, spinal inhibitory interneurons expressing neuropeptide Y (NPY) suppress this excitatory signaling, preventing innocuous touch from being perceived as an itch. These inhibitory interneurons are modulated by inputs from Merkel cell-associated Aβ-LTMRs. In old mice, the loss of Merkel cells impairs the inhibitory spinal pathway, leading to reduced suppression of mechanical itch signaling and consequently heightened mechanical itch sensitivity.

This study demonstrated a significant age-related decline in pruritogen-induced scratching behavior in old compared to young mice. This was shown by decreased responsiveness of DRG neurons to pruritogens and a downregulation of TRPV1 and Cav3.2 expression (Figure 6). The findings suggest dysfunction within the peripheral sensory system contributes to altered itch perception, which is commonly reported in older individuals. All six tested pruritogens elicited weaker scratching responses in old compared to young mice, whereas the control group, which received saline, showed no significant change (Figure 1). SLIGRL-NH_2_, a synthetic peptide that activates PAR2 and MrgprC11, induced the most pronounced age-related difference. This suggests that aging may disproportionately affect non-histaminergic signaling pathways. Activation of PAR2 sensitizes TRPV1 and TRPA1 via downstream GPCR pathways, amplifying itch sensation [18,19]. MrgprC11 potentiates itch by modulating TRPV1 activity [20,21]. Declined receptor signaling or downstream transduction (e.g., TRP channel activity) may be responsible for this age-related change.

At the neuronal level, calcium imaging revealed a reduction in the excitability and number of responsive DRG neurons in aged mice. Fewer neurons from old mice responded to either histamine or chloroquine, and none to both, in contrast to young mice (Figure 3). Although peak calcium amplitudes did not significantly differ between old and young mice, the reduced proportion of responsive neurons and decreased reactivity to Hi-K^+^ depolarization indicate impaired sensory neuron function with age. This aligns with previously reported studies showing age-associated increases in membrane excitability and impaired calcium dynamics in sensory neurons [22]. Although our findings point to age-related declines in pruritogen responsiveness, we cannot exclude contributions from non-specific factors such as neuronal loss, altered membrane properties, or reduced viability. For instance, if aged DRG neurons exhibit globally impaired excitability, the reduced response to pruritogens may reflect a broader functional decline rather than selective deficits in itch signaling. Future work combining electrophysiology, neuronal enumeration, and subtype-specific markers will help disentangle these possibilities.

One mechanistic contributor may be the observed decline in TRPV1 and Cav3.2 expression in DRG neurons from old mice (Figure 4 and Figure 5). TRPV1 is vital for transducing thermal, chemical, and inflammatory stimuli, while Cav3.2 facilitates low-threshold calcium influx involved in neuronal firing. Reduced expression of these channels may dampen action potential generation and synaptic transmission in pruriceptive circuits. These molecular changes are consistent with other age-related changes, such as reduced neurotrophic factor availability (e.g., NGF), mitochondrial dysfunction, and oxidative damage, which all impair ion channel expression and function [23,24,25,26]. Although chemical itch responses were diminished in pruritogen-injected old mice (Figure 1), mechanical itch and alloknesis were elevated in the naïve state (Figure 2), also consistent with a previous study [27]. This may reflect heightened excitability of certain sensory fibers or disinhibition at the spinal level due to age-related loss of GABAergic tone [28]. Mechanical itch can be mediated by activating Toll-like receptor 5 (TLR5)-positive Aβ low-threshold mechanoreceptors (Aβ-LTMRs), which convey light touch stimuli and are implicated in alloknesis. Notably, the mechanosensitive ion channel Piezo2, predominantly expressed in cutaneous Merkel cells, is a canonical receptor for light touch sensation. In aging, the decline in Merkel cell numbers may disrupt the modulation of tactile signaling by affecting the activity of both NPY^+^ and NPY^−^ inhibitory interneurons in the spinal cord [29]. After pruritogen injection, the increase in mechanical alloknesis was less robust in older animals, suggesting a limited capacity for peripheral sensitization or central amplification in the aged nervous system (Figure 2b,c and Figure 6). These contrasting findings, i.e., elevated baseline mechanical sensitivity yet reduced chemical itch, highlight the complexity of age-related sensory remodeling. It is possible that compensatory adaptations in the spinal cord or brain modulate the perception of different types of itch.

Further studies are warranted to investigate the molecular mechanism(s). Studies may include the investigation of central mediators such as gastrin-releasing peptide, natriuretic polypeptide B (NppB), and their downstream circuits, which are essential for itch-specific signaling in the spinal cord. Additionally, investigating age-related changes in inhibitory neurotransmitters through immunohistochemistry and pharmacologic modulation may elucidate which spinal mechanisms contribute to mechanical hypersensitivity in aged individuals. Collectively, this study identifies a distinct sensory phenotype in old mice characterized by reduced chemical itch sensitivity and altered expression of transduction molecules in DRG neurons. These findings contribute to a deeper understanding of how aging affects peripheral and central itch pathways and suggest potential molecular targets for alleviating chronic pruritus in the elderly.

## 5. Conclusions

In conclusion, the reduced itch responses observed in old mice, along with diminished DRG calcium signaling and lower expression of TRPV1 and Cav3.2, highlight an age-dependent decline in peripheral sensory transduction. These results improve our understanding of how aging affects itch perception and point to potential molecular targets for alleviating pruritus in older individuals.

## Data Availability

No data are available for this study.
